# The CHRS Data Portal, an easily accessible public repository for PERSIANN global satellite precipitation data

**DOI:** 10.1038/sdata.2018.296

**Published:** 2019-01-08

**Authors:** Phu Nguyen, Eric J. Shearer, Hoang Tran, Mohammed Ombadi, Negin Hayatbini, Thanh Palacios, Phat Huynh, Dan Braithwaite, Garr Updegraff, Kuolin Hsu, Bob Kuligowski, Will S. Logan, Soroosh Sorooshian

**Affiliations:** 1 Center for Hydrometeorology & Remote Sensing (CHRS), HSSoE, University of California, Irvine, CA, USA; 2 Department of Water Management, Nong Lam University, Ho Chi Minh City, Vietnam; 3 NOAA Center for Satellite Applications and Research (STAR), Maryland, USA; 4 International Center for Integrated Water Resources Management (ICIWaRM), Institute for Water Resources, US Army Corps of Engineers, Washington, D.C., Virginia, USA

**Keywords:** Water resources, Hydrology, Databases

## Abstract

The Center for Hydrometeorology and Remote Sensing (CHRS) has created the CHRS Data Portal to facilitate easy access to the three open data licensed satellite-based precipitation datasets generated by our Precipitation Estimation from Remotely Sensed Information using Artificial Neural Networks (PERSIANN) system: PERSIANN, PERSIANN-Cloud Classification System (CCS), and PERSIANN-Climate Data Record (CDR). These datasets have the potential for widespread use by various researchers, professionals including engineers, city planners, and so forth, as well as the community at large. Researchers at CHRS created the CHRS Data Portal with an emphasis on simplicity and the intention of fostering synergistic relationships with scientists and experts from around the world. The following paper presents an outline of the hosted datasets and features available on the CHRS Data Portal, an examination of the necessity of easily accessible public data, a comprehensive overview of the PERSIANN algorithms and datasets, and a walk-through of the procedure to access and obtain the data.

## Introduction

The growth of the field of remotely sensed hydrometeorology has benefited the world with the first near-global analyses of rainfall distribution. The quasi-global scope of these satellite-based precipitation products makes them practical for quantifying rainfall measurements over space and time, notably in regions that lack a large-scale system of precipitation gauges or a radar network—areas such as large remote sections of Africa, Asia, South America, and over the oceans. While remotely sensed hydrometeorological datasets have great utility, they suffer from a couple of significant drawbacks. First, the estimating methods that produce them are still in the development stage and are continuously undergoing considerable advancements. Second, the northern and southern latitudes of approximately 60° bound most quasi-global products owing to the increasing unreliability of satellite data readings as geostationary orbiting (GEO) satellites scan nearer to the poles.

In 1997, researchers from the University of Arizona—now situated at the Center for Hydrometeorology and Remote Sensing (CHRS) at the University of California, Irvine—developed one of the first satellite precipitation estimating systems, Precipitation Estimation from Remotely Sensed Information using Artificial Neural Networks (PERSIANN). As its name indicates, PERSIANN uses the machine learning technique known as artificial neural networks (ANNs) to determine the relationship between remotely sensed cloud-top temperature, measured by long-wave infrared (IR) sensors on GEO satellites, and rainfall rates, with bias correction from passive microwave (PMW) readings measured by low Earth-orbiting (LEO) satellites^1,2^.

From the framework of the PERSIANN algorithm, scientists at CHRS created two additional hydrometeorological estimation algorithms: PERSIANN-Cloud Classification System^3^ (CCS) and PERSIANN-Climate Data Record^4^ (CDR). PERSIANN-CCS estimates global rainfall in near real-time and at higher spatial resolution than PERSIANN through its cloud clustering algorithm and its use of IR data as the sole input. PERSIANN-CDR employs the PERSIANN algorithm with historical IR data starting in 1983, the first year that scientists regularly recorded remotely sensed data with a global span, to construct a consistent dataset for climate trend studies. In general, climate studies require at least 30 years of data for apparent trends to be deemed statistically viable^4^. We have applied Creative Commons CC0 open data licenses to all three datasets to ensure unimpeded free public distribution for academic and commercial purposes.

Gridded precipitation products like PERSIANN are useful in the research, community, and private sectors for hydrologic modeling, flood and drought prediction, water resource management, urban planning, and more. The need to make these exceedingly useful products available around the globe and ensure that researchers, professionals, educators, and community leaders get precisely the data they want is what led researchers at CHRS to construct the CHRS Data Portal. The CHRS Data Portal (https://chrsdata.eng.uci.edu) is a free-to-access data repository built in 2016 by a team of scientists and computer programmers at CHRS, with assistance from the United Nations Educational, Scientific, and Cultural Organization-International Hydrological Program (UNESCO-IHP) and the US Army Corps of Engineers International Center for Integrated Water Resources Management (ICIWaRM). The founders envisioned an open, easy-to-use, online web system where users can quickly customize, visualize, compare, and retrieve data from the three PERSIANN algorithms for analysis, modeling, and public education. Data subscriptions ensure that users of PERSIANN-family products always have up-to-date, customized data.

The following manuscript covers

- An overview of the datasets and tools available for use on the CHRS Data Portal.
- An assessment of the effects that CHRS’s datasets and web systems have had in the scientific community to date.
- A discussion on the need for easily accessible public data, especially in the field of satellite hydrometeorology, including its influence in further studies in the field and usefulness in public education.
- A detailed analysis of the available PERSIANN-family datasets including the algorithms that created them, the satellite-retrieved data used in their formulation, their spatiotemporal spans and characteristics, and a brief discussion on the strengths, weaknesses, and best applications of each algorithm.
- A concise description of the steps users of the CHRS Data Portal can use to operate the site, access the data, and utilize all available tools hosted on the webpage. This segment includes a brief walk-through of an example query.

## Results

### Data Repository Contents

The spatiotemporal characteristics of the global regular-interval satellite rainfall data (in mm) for the three PERSIANN products are accessible from the CHRS Data Portal web system, dependent on data availability. The principal utility of the CHRS Data Portal is to prepare customized data queries rapidly by aggregating and clipping PERSIANN data by using the query tools provided on the web system. Data products are customizable in their spatiotemporal extent and temporal resolution and are available for visualization, download, comparison, and subscription. These queries are performed directly on the interactive Google basemap that encompasses a large area of the web system. To visualize rainfall layers, the CHRS Data Portal uses the MapServer platform. MapServer is an open source platform that processes interactive maps on the web. MapServer was developed in the 1990s and is maintained at the University of Minnesota.

Users can find information on the PERSIANN algorithms with selected references on the production, validation, and improvements of the algorithms and studies on the philosophy of satellite precipitation estimation in the left-hand sidebar on the Data Portal webpage. The sidebar is split into three tabs, with each containing one PERSIANN-family algorithm’s information, namely a summary of the functions of the algorithm, details on when its data was last updated, its spatiotemporal scale and coverage, and links to download the original binary data files produced by the algorithm.

**Figure 1:**
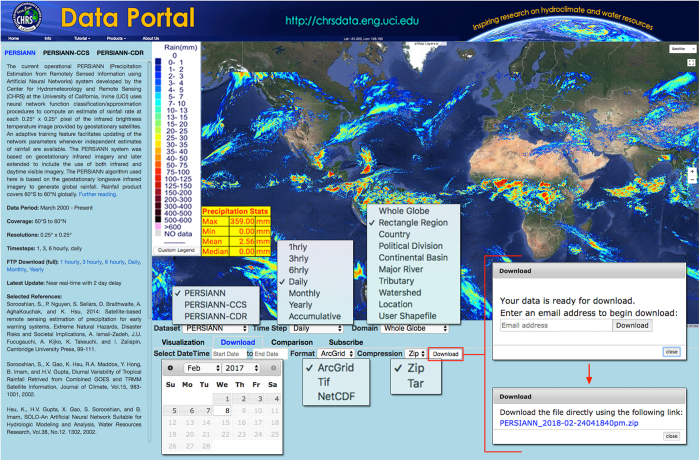
Overview of the CHRS Data Portal webpage. The drop-down menus and pop-ups displayed are characteristic of a Download query—indicated by the blue “Download” text near the top. Users may select other query types by clicking on the names of other tools that are horizontally in line with the Download tool. Note the customizable legend and the table of precipitation stats on the left side of the basemap, the information regarding the PERSIANN data in the left sidebar on the web system, and the menu items below the banner at the top of the page.

The toolbar on the top of the webpage includes a pop-up information sheet about the data repository; a narrated tutorial video that explores the full functionality of the CHRS Data Portal in depth; links to CHRS’s other web-hosted climate and weather data systems, iRain (http://irain.eng.uci.edu) and Rainsphere (http://rainsphere.eng.uci.edu); and a link to CHRS’s home page (http://chrs.web.uci.edu; Fig. 1).

### Datasets and Applications

PERSIANN-family datasets are utilizable for projects and research anywhere there is a demand for precipitation estimates over time and space between 60°N and 60°S. Due to the variations in spatiotemporal resolution, temporal extent, presence and method of bias correction, lag time for public availability due to processing, detection methods, and input sources, the decision of which dataset is best to employ for a project depends on the objective of the project. The original PERSIANN dataset represents the “middle of the pack”, with greater predictive skill than PERSIANN-CCS and a higher temporal resolution and with more rapid updates than PERSIANN-CDR. PERSIANN-CCS is best for high-resolution and real-time applications^5^, and PERSIANN-CDR is best utilized for projects over a lengthy timeline, principally for long-term trend analysis^4^. We outline the spatiotemporal resolution, temporal span, and the delay time owing to the preparation of each dataset in Table 1. We include further discussion on the benefits, drawbacks, and employment of each dataset in previous studies in the Methodology section.

In addition to the PERSIANN datasets, the CHRS Data Portal hosts numerous external precipitation datasets for comparison. The external datasets available for comparison are the NOAA National Centers for Environmental Prediction (NCEP) Climate Prediction Center (CPC) MORPHing technique^6^ (CMORPH), the NOAA/National Environmental Satellite, Data, and Information Service (NESDIS)-Hydro Estimator^7^ (HE), the Integrated Multi-satellitE Retrievals for GPM^8^ (IMERG), and the NOAA NCEP Stage IV radar and gauge product^9^ (Stage IV). CMORPH, HE, and IMERG are all satellite-based rainfall estimation datasets developed by NOAA and are some of the most employed datasets in the field. Additionally, Stage IV is included in the data repository as the “gold standard” comparison because of its high level of skill owing to the synthesis of gauge and radar measurements used in its production.

### Query Tools

Data from the PERSIANN family of algorithms is provided in various raster formats (ArcGrid, TIFF, and NetCDF) and is available for request on-site for instant download using the Download tool. Data requests for spatial coverages smaller than global scale come with ESRI shapefiles for easy importation into Geographic Information Systems (GIS) programs. Figure 1 summarizes the procedure for performing a Download query.

Besides being downloadable, any of the data from the PERSIANN family is applicable for direct visualization (raster data superimposed on the interactive Google basemap) using the Visualization tool. This tool also incorporates a table of generated characteristics that is displayed on the interactive map along with the mosaic rainfall data, a customizable legend, and superimposed political and hydrologic subdivision vectors.

**Figure 2:**
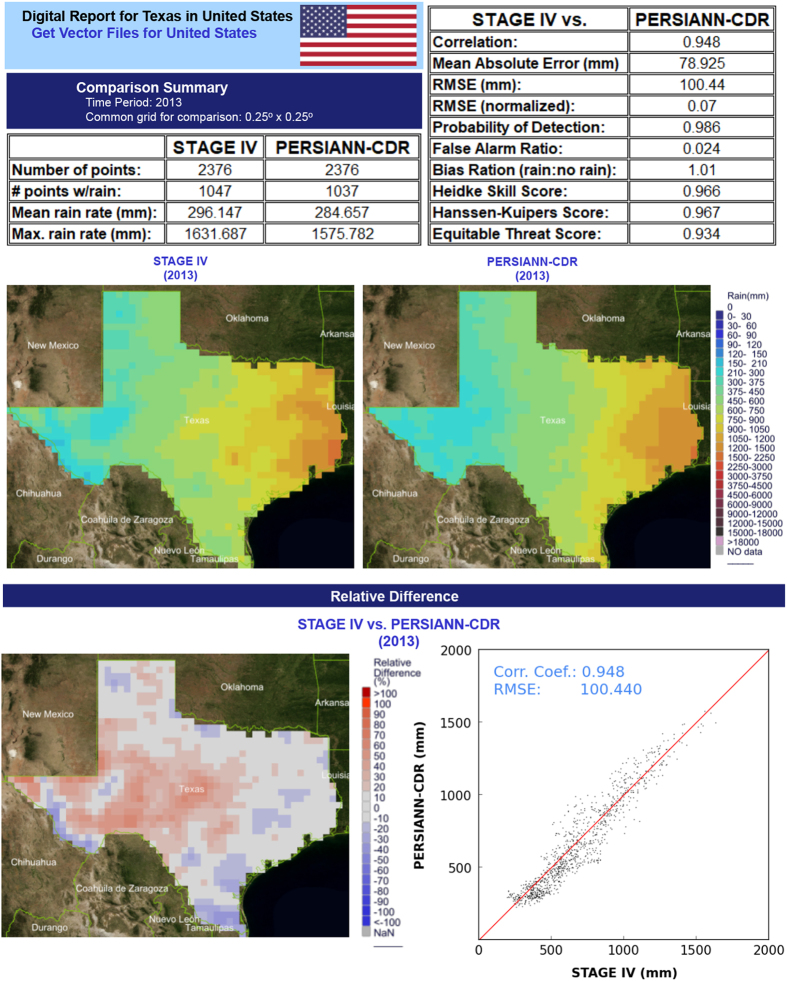
An example of the auto-generated reports created by the Comparison tool.

The Comparison tool compares PERSIANN-family datasets and other external datasets with matching time scales directly on the data repository. The tool generates a downloadable report of the characteristics of each dataset and establishes direct numerical and graphic comparisons between the primary dataset and a set of the comparison datasets (Fig. 2). These reports provide quick statistical assessments of the comparative skills of the queried datasets. Since the spatial resolutions of the three PERSIANN products and the comparison datasets vary, cubic kernel interpolation is performed prior to comparison to modify the spatial resolution of all datasets to 0.25°, which is the coarsest spatial resolution among all products. Characteristics and statistics prepared by the tool include the overall number of grid cells, the number of grid cells with predicted precipitation, mean rain rate (in units of mm per user-specified temporal resolution, hereby referred to as mm/T), maximum rain rate (in mm/T), plus various continuous and categorical metrics. Continuous metrics generated by CHRS Data Portal are the correlation coefficient (CORR), the mean absolute error (in mm), and the root-mean-square error (RMSE; normalized or in mm). Categorical indices produced by the CHRS Data Portal are the probability of detection (POD), the false alarm ratio (FAR), the bias ratio, the Heidke Skills score^10^, the Hanssen-Kuipers Score^11^, and the Equitable Threat Score^12^.

This report was generated by comparing PERSIANN-CDR with Stage IV for the state of Texas as the spatial domain and the year 2013 as the time span. The CHRS Data Portal’s reports include a table of characteristics such as total number of pixels, number of pixels with rain, mean rain rate, maximum rain rate, and various statistical metrics (top). Additionally, maps of the accumulation of rainfall from the primary and comparison datasets (middle-left and middle-right, respectively) and comparison maps their relative difference (bottom-left) are generated in the report. Moreover, correlation graphs (bottom-right) are generated with CORR and RMSE values for further comparison. Not pictured are the generated maps and corresponding bar graphs of elevation, land use, and climate type over the same spatial domain.

The CHRS Data Portal offers a subscription database system intended to serve users who are interested in newly available data within a region of interest. Users interested in using the Subscribe tool can query a subscription by customizing location, product, spatiotemporal extent, and temporal resolution. The database system retrieves real-time data, processes it according to each user’s request(s), notifies subscribers, and provides customized data through temporary links. The main advantage of the subscription system is to free users from querying and checking data manually after every update. A step-by-step walk-through on the process of making a query and an example query are detailed in the Methods section.

All PERSIANN datasets come as gridded data with quasi-global spatial coverage, but users can delineate and aggregate data for all query types (*e.g.* location for subscription queries) via political borders, watershed boundaries, custom-defined rectangles, or defined by a shapefile. Shapefile formats utilizable by the CHRS Data Portal for custom data query boundaries are ESRI shapefile (.shp, .shx, and .prj), Keyhole Markup Language (.kml/.kmz), and GeoJSON (.geojson).

### Usage Statistics

The CHRS Data Portal has been under development for the last two years with no journal publications. Yet, as of June 27th, 2018, users from 145 different countries have accessed the site 41,879 times, downloading 13.9 terabytes of data. CHRS’s push to promulgate information about the database commenced only in 2018. Therefore, we foresee the number of visitors and the volume of data downloads to proliferate in the near future. The most in-demand data is from PERSIANN-CCS at 10.2 terabytes of total downloaded data (this is expected since PERSIANN-CCS has the densest spatial resolution), then PERSIANN at 2.2 terabytes of data requests, and lastly, PERSIANN-CDR at 1.5 terabytes of data downloads. Our primary source of traffic is from the United States, accounting for over half the traffic. Other countries with notable visitor totals include Iran, with about 2,400 unique visits; India, with around 1,500 unique visitors; and China and Peru, both with approximately 1,200 visits each.

One of the metrics we may employ to quantify the influence of the PERSIANN family of algorithms in the academic sector, and by extension derive a notion of the potential impact further awareness of the CHRS Data Portal may have, is the total number of citations each algorithm’s original journal articles have. In this case, we are employing (1) the original paper on the formulation of the algorithm and (2) the study with the first evaluations of the products from each algorithm. PERSIANN^1,2^ has been cited 1,415 times since 1997, PERSIANN-CCS^3,13^ has been cited 480 times since 2004, and PERSIANN-CDR^4,14^ has been cited 239 times since 2015, for a sum of 2,134 citations for the entire PERSIANN family.

## Discussion

The CHRS Data Portal creates a synergistic relationship between data providers and data consumers by providing easy public access of the PERSIANN-family datasets to a broad spectrum of users. The new CHRS Data Portal can support and influence future investigations in climatology, remote sensing, estimation of precipitation for early warning systems, extreme natural hazards, disaster risks, and more^15^. These future studies can, in turn, enhance the consistency, skill, coverage, and timeliness of high-resolution precipitation estimation and forecasting algorithms. Additional studies on the reliability of hydrologic predictions based on satellite-derived precipitation data are crucial due to the multitude of estimation approaches and the prevailing uncertainties in retrieving precipitation characteristics from satellite information^16–18^. Improvements in precipitation frequency and accuracy and the deterioration of *in-situ* global systems for hydrologic measurements^19,20^ are the catalysts behind these investigations.

One of the objectives of the CHRS Data Portal is to prioritize public availability and convenience, with the goal of promoting the utilization of PERSIANN-family data beyond research purposes. One of the most crucial issues in virtually all scientific disciplines is understandably and engagingly conveying the results and implications of significant studies. In response to this, CHRS has established community education on the science and impacts of precipitation as one of its fundamental missions. This focus on public education contributed to the release of the CHRS Data Portal, along with the GIS-based iRain and RainSphere web systems. Each of these websites emphasizes presenting data in a captivating and easily digestible form, with the use of colorful representations of mosaic data, simple-to-understand tools, measurements and comparisons done automatically on the web system, and informational videos on each website that make understanding and maneuvering around the systems’ features straightforward.

## Methods

**Figure 3:**
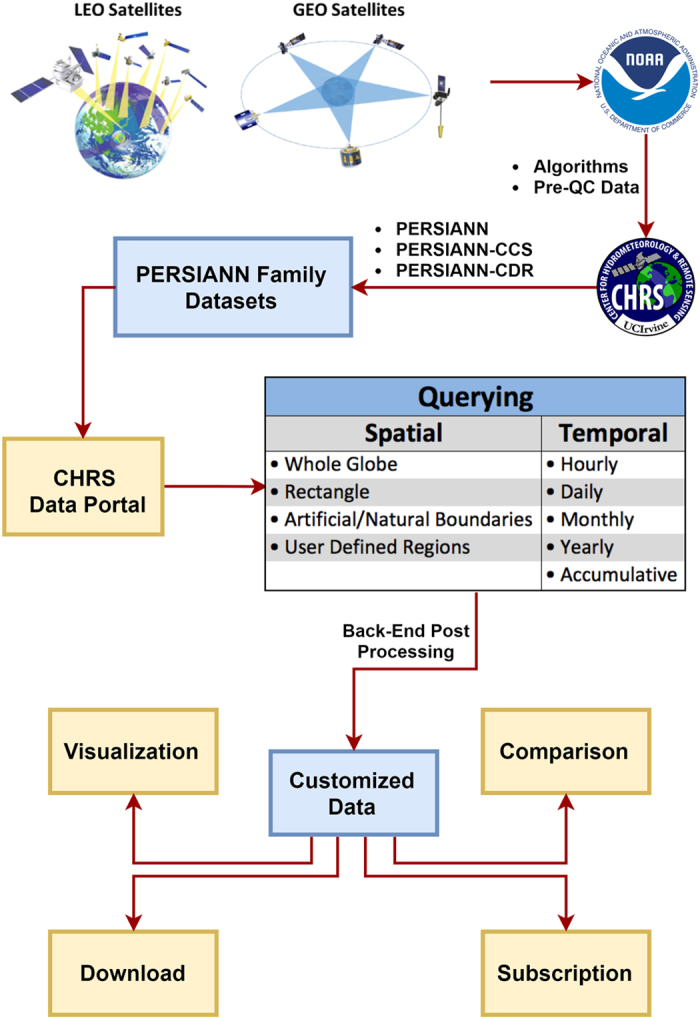
Flowchart from remote sensed data to user query. This flow chart summarizes all the processing that encompasses queries done on the CHRS Data Portal. First, raw data is captured using GEO and LEO satellites. Then, it is processed on servers at NOAA and CHRS to produce the PERSIANN family of datasets. After a user queries a chosen dataset for a given spatiotemporal extent and temporal resolution, the data is processed in the back end of the data repository’s system. Finally, the customized data query is delivered to the user in a manner dependent on the tool that was chosen.

This section describes the minutiae of the overarching process of making a custom data query, beginning with the acquisition of input data, then the generation of PERSIANN datasets, and concludes with a detailed walk-through of how to operate the CHRS Data Portal, including a supplemental example query. Special attention is given to the production and properties of the PERSIANN datasets to ensure that readers interested in their use can make informed decisions. Figure 3 illustrates the operating system of the CHRS Data Portal including all the processes from raw data acquisition to processing of user queries.

### The Development and Utility of the PERSIANN Datasets

IR satellite imagery is the primary input for developing the three PERSIANN-family datasets. NOAA’s NCEP CPC provides IR imagery for PERSIANN and PERSIANN-CCS while PERSIANN-CDR uses NOAA’s NCEP GridSat-B1 dataset^21^. While CHRS’s servers produce PERSIANN and PERSIANN-CDR by implementing the algorithms locally, NOAA NESDIS’s server generates PERSIANN-CCS data with an approximate lag time of one-hour, which is referred to as real-time PERSIANN-CCS. After two days, real-time PERSIANN-CCS is replaced by PERSIANN-CCS data processed at CHRS using IR data from CPC and NCEP.

PERSIANN is the original precipitation product from CHRS that operates primarily by utilizing an adaptive ANN to estimate precipitation. Hsu and coauthors initially calibrated the ANN over the Japanese Islands by using precipitation data produced from precipitation gauges and radars from the Automated Meteorological Data Acquisition System (AMeDAS) developed in Japan. By recursively revising the model parameters upon the availability of ground-based data, the PERSIANN product performed “surprisingly well” in capturing rainfall over various regions and periods^1^.

PERSIANN has established its niche as the “intermediate” choice among the products: coarser in spatial resolution and with a more extended delay period between releases compared to PERSIANN-CCS and smaller in temporal duration compared to PERSIANN-CDR, but with greater accuracy than PERSIANN-CCS and with more rapid updates than PERSIANN-CDR. PERSIANN’s greatest virtues over its near real-time counterpart PERSIANN-CCS are the implementation of PMW data for bias correction, which enhances its detection of warm and orographic precipitation, and its longer temporal span. PERSIANN data is best employed for projects that call for short-delay input with a high level of accuracy, such as identifying flood hazard risk in data-sparse regions^22^, use as training data for climate models, use as input data for hydrologic systems, analysis in short-duration climatology studies, utilization for recurrence interval analysis, and so forth.

The PERSIANN-CCS algorithm provides high-resolution precipitation in near real-time through its near-instantaneous input source and unique architecture. PERSIANN-CCS operates by segmenting clouds into patches by utilizing morphological and watershed segmenting techniques, then clusters cloud patches into cloud “types” based on coldness, geometry, and texture via k-means clustering, and finally trains cloud-top temperature and rainfall relationships characteristic for each cloud type. The greatest assets of PERSIANN-CCS are the brief time span between data retrieval to output, which can assist in quickly modeling and predicting floods and have applications for water resource management, and its rich spatial resolution, which is approximately 39 times denser with information than the resolution of PERSIANN and PERSIANN-CDR. PERSIANN-CCS’s most significant shortcomings come from its absence of bias correction and its use of solely one type of input data. This results in PERSIANN-CCS’s data being less accurate than the other PERSIANN products that utilize bias correction, notably for warm rains and highlands^13^.

PERSIANN-CCS data is sufficient for high-resolution, near real-time precipitation analyses, notably in remote sectors of the world where an extensive gauge or radar network for precipitation data retrieval may not exist, such as in the examination of Typhoon Haiyan over the Philippines^23^. PERSIANN-CCS has also been used for establishing a universal early warning system^24^, assisting with flood forecasting and inundation mapping for a case study in Iowa^25^, forecasting of short-term object-based precipitation^26^, and simulating soil moisture^27^. PERSIANN-CCS data is further accessible via a user-friendly visual display on CHRS’s iRain website.

PERSIANN-CDR’s primary purpose is for use in climate analysis, specifically for trend analysis. PERSIANN-CDR employs IR satellite data (10 km spatial and 3-hourly temporal resolutions) from the NOAA International Satellite Cloud Climatological Project (ISCCP), dating back to 1979, to produce an archive of precipitation data. As opposed to the PERSIANN algorithm, which updates the ANN’s parameters recursively, PERSIANN-CDR uses a fixed array of ANN parameters trained from hourly Stage IV data. The Global Precipitation Climatology Project^28–30^ (GPCP) precipitation product is available in monthly temporal resolution and 2.5° spatial resolution and was incorporated into the PERSIANN-CDR archive to diminish bias. PERSIANN-CDR’s most significant shortcomings are the long periods between updates and its coarser temporal resolution.

Some recent examples of conducted research projects using PERSIANN-CDR include, but are not limited to, global precipitation change assessment across spatial scales^31^, drought monitoring over a semiarid region^32,33^, characterizing precipitation patterns in the southern part of the Amazon basin^34^, rainfall frequency analysis in Colorado and California, USA^35^, and predicting heat waves in Bangladesh^36^. Moreover, trend analysis from PERSIANN-CDR can be used to select an appropriate Coupled Model Intercomparison Project 5 (CMIP5) model for projection of future trends in precipitation intensity and extent^37^. As with PERSIANN-CCS, PERSIANN-CDR data is likewise accessible via a user-friendly visualization on CHRS’s RainSphere website^38^.

Since remotely sensed precipitation estimation is still a relatively young area of research, many of its products suffer from considerable inaccuracies owing to limitations such as unsolved problems in the field and lack of information-rich input data. As the field progresses, so do the PERSIANN family of datasets. Improvements of the datasets are made by integrating multispectral satellite information^39^ and by incorporating PMW information from LEO satellites^40^ for bias correction. Furthermore, investigations on the effectiveness of utilizing cloud segmenting techniques, deep learning methods, integrating climatology and topography effects, and the addition of newer satellites like Geostationary Operational Environmental Satellites (GOES)-R and GOES-S and new datasets like NOAA Multi-Region, Multi-Satellite (MRMS) are being done continuously by the researchers at CHRS. On the horizon, CHRS scientists are working hard to produce the 2nd generation of PERSIANN datasets from these investigations.

### User Queries

The CHRS Data Portal was created to facilitate universal usability as its main priority. Therefore, researchers at CHRS have simplified the process of querying the data for use to as few steps as possible, while ensuring the data is available in the most accessible data formats. The steps required for each query follow the same structure and are outlined in the following tool-specific subsections: Subscription, Visualization, Comparison, and Download. Users should refer to Figs 1 and 3 while viewing the subsequent sections to assist with comprehension.

### Subscription

(1) A PERSIANN-family dataset must be chosen from the “Dataset” drop-down menu.
(2) A time step for the data is specified. Time steps available depend on the algorithm’s temporal resolution.
  - For PERSIANN and PERSIANN-CCS: 1-hourly (“1 hrly”), 3-hourly (“3 hrly”), and 6-hourly (“6 hrly”)
  - For all PERSIANN products: daily, monthly, yearly, and accumulative
(3) A spatial domain for the data is chosen among global extent or various political, hydrologic, and user-defined boundaries.
  - Political boundaries: country and “political divisions” (administrative divisions of a country, *e.g.* states, provinces, oblasts, etc.)
  - Hydrologic boundaries (listed in descending magnitude of size): continental basin, major river basin, tributary basin, and watershed
  - User-defined boundaries: rectangle (drawn by the user directly onto the interactive map), custom shapefile (.shp + .shx + .prj, kml/kmz, geojson), and location (pixel query)
(4) The user chooses the Subscribe tool, inputs their email address, and hits “Subscribe”.

### Visualization

Steps 1–3 are identical to steps 1–3 from the Subscribe subsection.

(4) The user should choose the Visualization tool.
(5) A temporal span must be specified from the “DateTime” menu.

### Comparison

Steps 1–5 of using the Comparison tool are the same as the steps 1–5 of the Visualization tool section, except for the Comparison tool being chosen instead of the Visualization tool in step 4.

(6) The next step is to choose which dataset(s) they will use to make statistical comparisons. Datasets include the PERSIAN-family products, CMORPH, IMERG, HE, and Stage IV.

Figure 2 shows one example of an auto-generated report created using the Comparison tool.

### Download

Steps 1–6 of using the Download tool are the same as the steps 1–6 listed in the Comparison tool section, except for the selection of the Download tool in step 4 and choices limited to only PERSIANN-family products in step 6.

(7) The user querying the data needs to specify (a) an output data format among ArcGrid, Tif, and NetCDF and (b) a compression type between Zip and Tar.
(8) Press the “Download” button and enter your email address as needed for larger data queries. Smaller data queries are available directly from the web system.

### Example Query

To illustrate, imagine that a user chooses to download PERSIANN data for each day during Hurricane Harvey’s duration over the state of Texas and implement it in a GIS program. To accomplish this, they would take the following steps in order: (1) choose “PERSIANN” from the “Dataset” menu; (2) pick “Daily” from the “Time Step” menu; (3) select “Political Boundary” from the “Domain” menu; (4) click on the state of Texas in the interactive map; (5) select the Download tab; (6) specify August 25th, 2017 to August 31st, 2017 in the “Select DateTime” tab; (7a) pick “ArcGrid” from the “Format” option; (7b) decide on the appropriate compression type for their operating system from the “Compression” list; and (8) click on the “Download” button, which will pop up with an email address request. After the user enters their email, the compressed folder with their customized data will either be downloaded from the website or a link will be sent to their email, depending on the size of the request. A narrated video tutorial with more in-depth directions and walk-throughs is provided at the top of the website under the heading “Tutorial.”

## Additional information

**How to cite this article**: Nguyen, P. *et al*. The CHRS Data Portal, an easily accessible public repository for PERSIANN global satellite precipitation data. *Sci. Data*. 6:180296 doi: 10.1038/sdata.2018.296 (2019).

**Publisher’s note**: Springer Nature remains neutral with regard to jurisdictional claims in published maps and institutional affiliations.

## Figures and Tables

**Table 1:** Spatiotemporal characteristics of the PERSIANN products.

Datasets	Spatial Resolution	Spatial Coverage	Finest Temporal Resolution	Temporal Coverage	Delay Time
PERSIANN	0.25°×0.25°	Quasi-global (60°S–60°N; 180°W–180°E)	Hourly	March 1^st^, 2000 to present	2 days
PERSIANN-CCS	0.04°×0.04°	Quasi-global (60°S–60°N; 180°W–180°E)	Hourly	January 1^st^, 2003 to present	1 hour
PERSIANN-CDR	0.25°×0.25°	Quasi-global (60°S–60°N; 180°W–180°E)	Daily	January 1^st^, 1983 to present	3 months
